# Intra-aortic and Intra-caval Balloon Pump Devices in Experimental Non-traumatic Cardiac Arrest and Cardiopulmonary Resuscitation

**DOI:** 10.1007/s12265-022-10343-9

**Published:** 2022-12-08

**Authors:** Emanuel M. Dogan, Birger Axelsson, Oskar Jauring, Tal M. Hörer, Kristofer F. Nilsson, Måns Edström

**Affiliations:** 1 Department of Anesthesiology and Intensive Care, Faculty of Medicine and Health, Örebro University, SE-701 85 Örebro, Sweden; 2 Department of Cardiothoracic and Vascular Surgery, Faculty of Medicine and Health, Örebro University, Örebro, Sweden

**Keywords:** Heart arrest, Cardiopulmonary resuscitation, Counterpulsation, Hemodynamics

## Abstract

Intra-aortic balloon pump (IABP) use during CPR has been scarcely studied. Intra-caval balloon pump (ICBP) may decrease backward venous flow during CPR. Mechanical chest compressions (MCC) were initiated after 10 min of cardiac arrest in anesthetized pigs. After 5 min of MCC, IABP (*n* = 6) or ICBP (*n* = 6) was initiated. The MCC device and the IABP/ICBP had slightly different frequencies, inducing a progressive peak pressure phase shift. IABP inflation 0.15 s before MCC significantly increased mean arterial pressure (MAP) and carotid blood flow (CBF) compared to inflation 0.10 s after MCC and to MCC only. Coronary perfusion pressure significantly increased with IABP inflation 0.25 s before MCC compared to inflation at MCC. ICBP inflation before MCC significantly increased MAP and CBF compared to inflation after MCC but not compared to MCC only. This shows the potential of IABP in CPR when optimally synchronized with MCC.

**Graphical Abstract:**

The effect of timing of intra-aortic balloon pump (IABP) inflation during mechanical chest compressions (MCC) on hemodynamics. Data from12 anesthetized pigs.

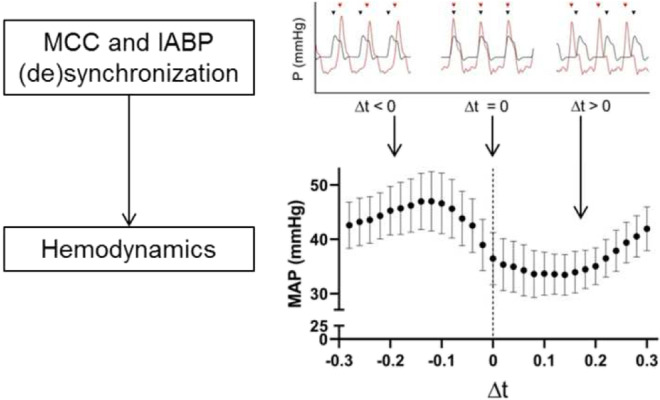

**Supplementary Information:**

The online version contains supplementary material available at 10.1007/s12265-022-10343-9.

## Introduction

Despite several decades of research in the treatment of out-of-hospital cardiac arrest, the survival rate remains low [1, 2]. Early cardiopulmonary resuscitation (CPR) has increased survival from 4 to 10%, highlighting the importance of early circulatory support [3]. Fundamental to improving survival is an understanding of the physiology and mechanism of blood flow and pressures during CPR. CPR was introduced in the 1960s [4], but the mechanism of blood flow still remains unclear. It is believed that coronary perfusion pressure (CPP) correlates with the rate of return of spontaneous circulation (ROSC) [5]. Different drugs to improve hemodynamic parameters have been studied, but the outcome is frequently disappointing [6].

Resuscitative endovascular balloon occlusion of the aorta (REBOA), as a part of the EVTM concept (endovascular resuscitation and trauma management) [7], is used to achieve continuous occlusion of the aorta by endovascular means. REBOA has been shown to increase systemic blood pressure, CPP, and survival in both experimental CPR and case reports [8–12]. Its clinical effect during non-traumatic cardiac arrest is now being studied [13]. However, obstruction of aortic blood flow throughout the whole CPR cycle increases the ischemic burden distal to the occlusion. Possibly, periodic aortic occlusion synchronized with the mechanical compression cycle may be sufficient to replicate the beneficial effects of REBOA on blood pressure and forward flow.

The intra-aortic balloon pump (IABP) is set to inflate during diastole and deflate before systole in spontaneous circulation and is the most commonly used circulatory support device in cardiogenic shock patients [14]. The “diastolic augmentation” increases diastolic blood pressure (DBP), CPP, and, subsequently, myocardial blood flow and oxygen supply. The “systolic unloading” reduces the workload of the ventricles by reducing the afterload, which in turn reduces the myocardial oxygen demand [15–17]. However, routine use of IABP in cardiogenic shock patients is not supported by large studies [18–21].

The effect of IABP during cardiac arrest and CPR is not well studied. An experimental study from 1988 showed that IABP increased CPP and DBP during mechanical CPR [22]. A case report from 2018 also demonstrated the beneficial effects of IABP during CPR [23]. However, the optimal timing of inflation/deflation of the balloon for blood pressure and flow augmentation in the CPR cycle has never been studied.

Furthermore, prevention of the suggested retrograde blood flow from the right atrium to the vena cava, occurring during CPR [24, 25], could be beneficial. Although systemic retrograde flow is in part prevented by venous valves at the thoracic inlet [24], no valves are present in the inferior vena cava [26]. Hypothetically, a periodically inflated balloon in the vena cava, an intra-caval balloon pump (ICBP), could prevent this pendular blood volume and deflation of the balloon during decompression could increase preload. To the best of our knowledge, no previous study has investigated a balloon pump in the vena cava during CPR.

The aim of this study was to explore the hemodynamic effects of balloon pump devices in either the aorta (*i.e*., manipulation of forward flow and pressure) or the vena cava (*i.e*., manipulation of preload and backward pressure and flow) in relation to the compression-decompression cycle during experimental CPR in anesthetized pigs. The primary hypothesis was that an IABP or ICBP, inflated during the compression phase and deflated during the decompression phase of MCC, would increase systemic arterial pressure (SAP) and carotid blood flow (CBF), and that the CPP would increase when IABP was inflated during the decompression phase. The primary outcomes of using balloon pump devices in both locations were SAP, CPP, and CBF, as measures of efficient output.

## Materials and Methods

### Animals

The Regional Animal Ethics Committee in Linköping approved the study (ID: 17,619–2020) prior to experimentation. Twenty pigs, both sexes, of Swedish country breed (Hampshire and Yorkshire, 3–4 months old with a mean weight of 30 kg [range 28–32 kg]) were included. The research protocol was performed by an experienced research team and was supervised by a licensed veterinarian. The experiments were performed over two weeks in February 2021 with two simultaneous experiments each day in a laboratory equipped for animal experimentation at Örebro University Hospital, Örebro, Sweden.

### Animal Preparation

Animal preparation has been recently described [9]. In short, the pigs were sedated at the farm with azaperone, transported to the laboratory, anesthetized, and medicated (induction of anesthesia: tiletamine, zolazepame, azaperone, atropine, cefuroxime; maintenance of anesthesia: propofol and remifentanil). The pigs were intubated, mechanically ventilated, Ringer’s acetate and 5% glucose were given to compensate for fluid losses, and euthanized by administering potassium chloride in general anesthesia at the end of the study.

### Surgical Preparations

**Fig. 1:**
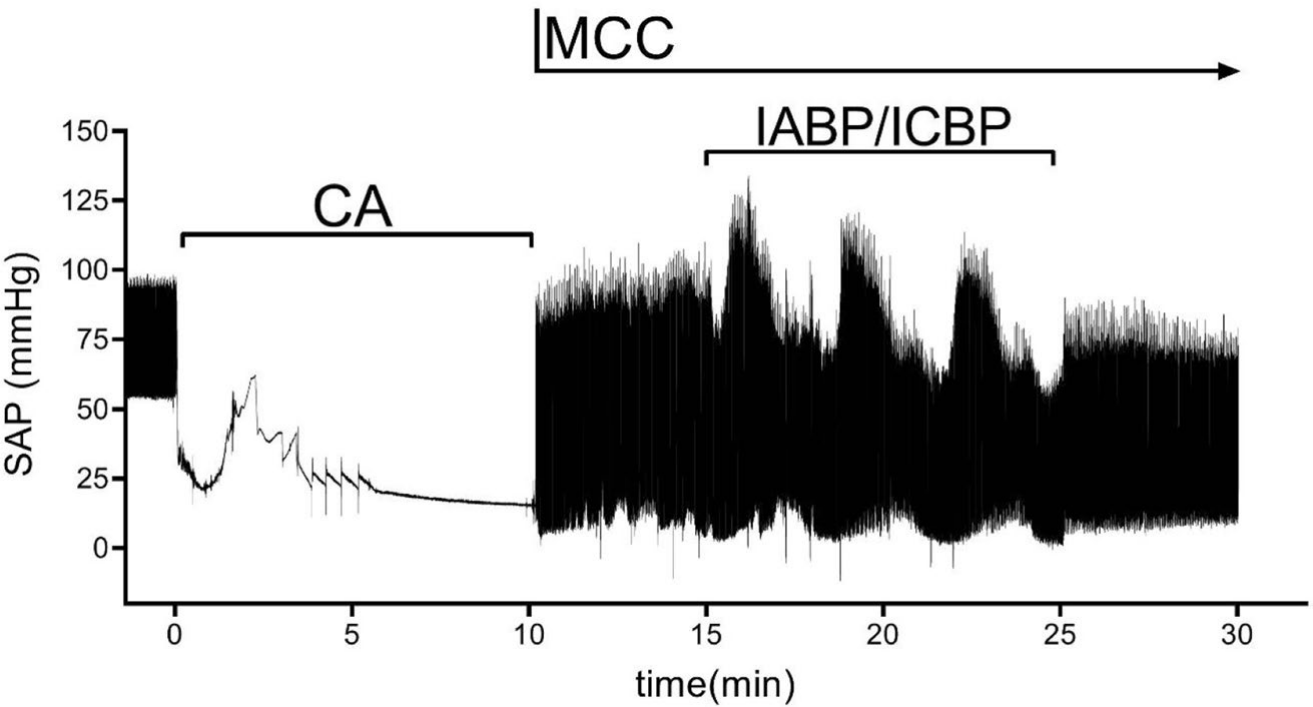
Experimental overview depicted by typical systemic arterial pressure (SAP) measurements in an anesthetized pig receiving mechanical chest compressions (MCC) after circulatory arrest (CA) with concurrent intra-aortic balloon pump (IABP). Note that time 0 on the *x*-axis shows the time for initiation of ventricular fibrillation; the time used for induction of anesthesia and instrumentation (at *x* < 0) has been cropped for illustration purposes. ICBP: intra-caval balloon pump

The animals were surgically prepared as has been recently described [9]. The following catheters were inserted: a right carotid introducer for systemic blood pressure measurements, an introducer in the right femoral artery for the IABP (Mega 8 Fr, 50 cc, Maquet, Getinge AB), an introducer in the right jugular vein for the bipolar pacemaker electrode used to induce ventricular fibrillation (VF) and a central venous line in the right jugular vein for the ICBP (Mega 8 Fr, 50 cc, Maquet, Getinge AB). Flow probes were attached around the left internal carotid artery and the left jugular vein for flow measurements. A minimal abdominal incision was performed to insert a 14 Fr urinary catheter into the urinary bladder. An intervention-free period of at least one hour followed the surgical preparations (Fig. 1).

### Protocol and Measurements

**Fig. 2:**
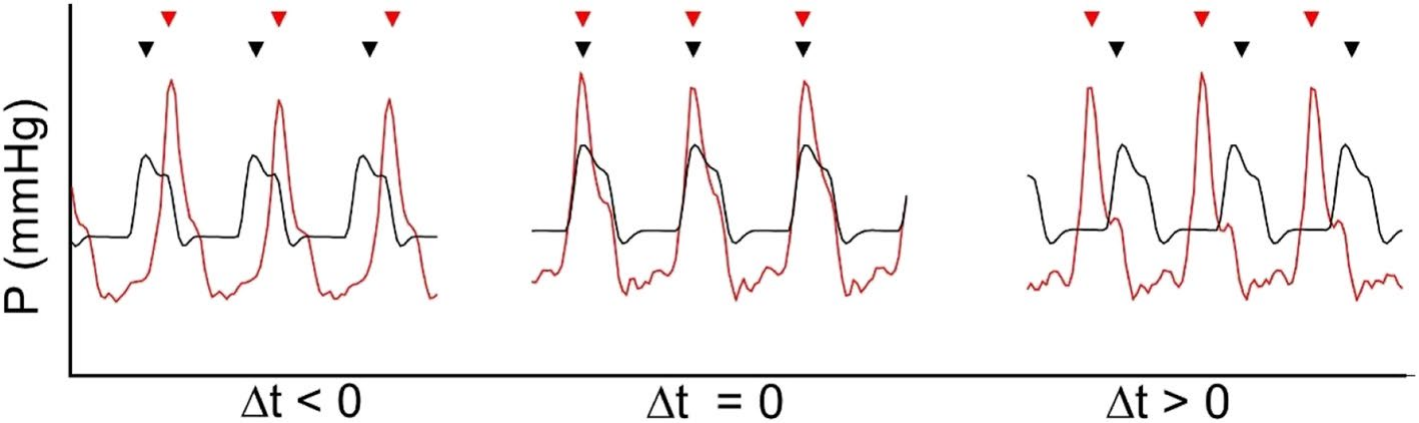
$\Delta t$ showing the offset in synchronization of IABP/ICBP inflation and MCCs. Typical data are presented and are normalized for illustrative purposes.  : IABP/ICBP,  : MCC

After surgical preparations and through the experiment, data were collected regarding blood flow, hemodynamic, and respiratory parameters (MP150; BioPac Systems, Goleta, CA). The first six animals were used for model development. VF was induced by applying a voltage of 9 V to the bipolar pacemaker electrode inserted in the right ventricle. When VF was confirmed by ECG readings, the ventilator and infusion pumps were disabled until the start of CPR. The duration of circulatory arrest was 10 min. Mechanical chest compressions (MCC) were initiated using a chest compression device (LUCAS™, JOLIFE AB, Lund, Sweden). Ventilation was restarted with 10 breaths per minute and tidal volumes of 6 ml/kg. MCC continued for 5 min before the IABP or ICBP was initiated. The IABP was placed in the proximal thoracic aorta, and the ICBP was placed, covering the confluence of the superior and inferior vena cava in the right atrium. The balloon pump continued for 10 min before a 5-min period with only MCC (Fig. 2). The frequencies of MCC and IABP/ICBP inflation differed slightly, resulting in a progressive phase shift between the devices during the periods of simultaneous MCC and IABP/ICBP. This progressive phase shift was later used in the data analysis (see below). The baseline was defined as the initial period of MCC.

### Data Processing and Variables

Data from the period of simultaneous MCC and IABP/ICBP was extracted using AckNowledge (Biopac Systems), including IABP/ICBP pressure, SAP, central venous pressure (CVP), CPP, CBF, and jugular vein blood flow (JBF). CPP was calculated as $CPP=DBP-CVP$, where diastolic blood pressure (DBP) was defined as SAP during MCC decompression. Systemic arterial peak pressure was used as a surrogate for MCC timing, and MCC peak pressure refers to peak SAP during MCC. IABP/ICBP peak pressure was used to assess IABP/ICBP inflation. SciPy for Python 3.8.10 was used for data processing [27]. Mean arterial pressure (MAP) and mean carotid and jugular blood flow (mCBF and mJBF) were calculated as the average sum of the measurements between two consecutive positive peaks. For all variables, positive peaks without a following negative peak and negative peaks without preceding positive peaks were discarded. Peak outliers (defined as a *z*-score ≥ 5) were removed.

### Progressive Phase Shift of IABP/ICBP and MCC

Initial measurements of the MCC during method development showed a frequency of 102 compressions/min (frequency of 1.7 Hz). IABP/ICBP inflation frequency was therefore set to 102 inflations/min. During a more thorough analysis, we found that the mean frequency of IABP/ICBP inflation was 1.7001 (SD 7.24 × 10^−5^) Hz and the mean frequency of MCC was 1.6949 (SD 2.10 × 10^−4^) Hz, resulting in a progressive phase shift between IABP/ICBP inflation and MCC. The sampling rate of MP150 (Biopac Systems) was 50 Hz (*i.e.*, the time resolution was 0.02 s). During the 10-min period with simultaneous MCC and IABP/ICBP, the time difference between MCC and IABP/ICBP inflation was calculated per peak ($\Delta t={t}_{IABP/ICBP}-{t}_{MCC}$), where MCC was associated with the temporally closest IABP/ICBP positive pressure peak, before or after. $\Delta t<0$ designates IABP/ICBP inflation before MCC peak pressure, $\Delta t>0$ IABP/ICBP inflation after MCC, and at $\Delta t=0$ inflation and compression occur simultaneously (Fig. 2). Based on the frequencies of MCC and IABP/ICBP and the resulting progressive phase shift, possible values for $\Delta t$ was between $-$ 0.28 and 0.30 s. For each variable, there were 25–35 samples for every $\Delta t$ (*i.e.*, every phase shift of 0.02 s between IABP/ICBP and MCC) over the period of simultaneous MCC and IABP/ICBP (IABP/ICBP in Fig. 1). A declining trend of hemodynamic variables over time in animals with IABP was noted (Supplementary Fig. S1), and therefore, only data from the first beat frequency period was used, unless otherwise stated, comprising 8–12 samples per variable for every $\Delta t$, both for IABP and ICBP experiments. To investigate the hemodynamic effects of different peak IABP and peak MCC time differences, optimal ($\Delta {t}_{\mathrm{optimal}}$) and least optimal, unfavorable $\Delta t$ ($\Delta {t}_{\mathrm{unfav}.}$), defined as the maximum and minimum mean outcome variable value over the range of $\Delta t$, respectively, were identified for all variables.

### Statistical Methods

The primary outcomes of SAP, CPP, and CBF for both IABP and ICBP interventions were the dependent variables. The effect of different $\Delta t$ (independent variable) on the dependent, primary outcome variables was investigated. Three different $\Delta t$ were compared using a repeated-measures ANOVA; optimal $\Delta t$ ($\Delta {t}_{\mathrm{optimal}}$), least optimal, unfavorable $\Delta t$ ($\Delta {t}_{\mathrm{unfav}.}$), and baseline MCC (*i.e.*, without IABP/ICBP). *p* ≤ 0.05 was considered significant, and *p* ≤ 0.1 denotes a trend. Data are presented as mean values and standard deviation (SD) or standard error (SE), where appropriate. All statistical analyses were performed in SPSS v23 (Armonk, NY, USA). Balloon inflation timing (different $\Delta t$) and baseline values (MCC only) were compared with repeated-measures ANOVA.

## Results

The first 6 animals were used for model development. One animal died during surgical instrumentation due to bleeding. One animal was excluded due to ventilator malfunction; 12 animals were included in the final experimental protocol: 6 animals in the ICBP group and 6 animals in the IABP group.

### Intra-aortic Balloon Pump Experiments

#### Optimal $\Delta {{t}}$ for IABP and MCC

**Fig. 3:**
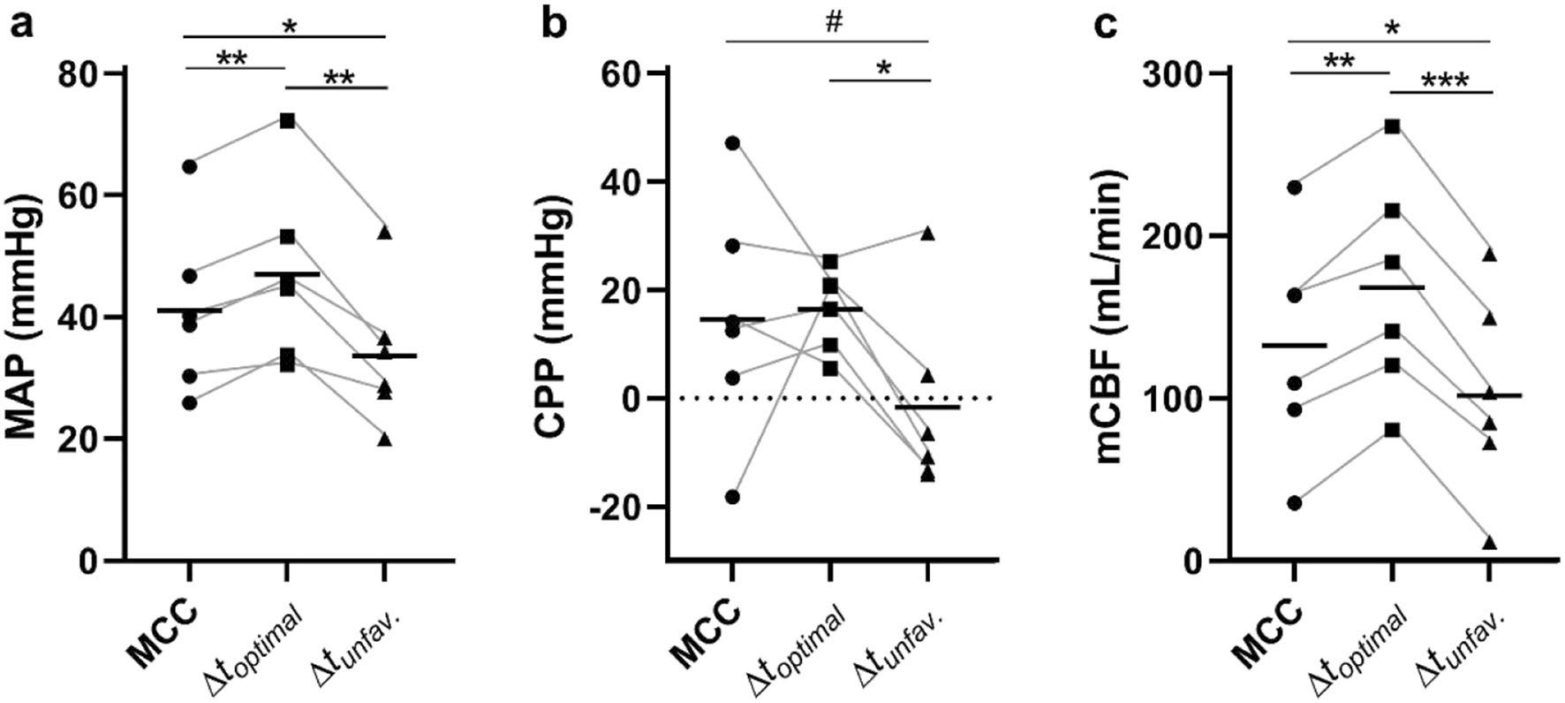
MAP (**a**), CPP (**b**), and mCBF (**c**) at baseline (MCC) without concurrent IABP, optimal $\Delta t$ ($\Delta {t}_{\mathrm{optimal}}$), and unfavorable $\Delta t$ ($\Delta {t}_{\mathrm{unfav}.}$) in anesthetized pigs (*n* = 6). Bars denote the sample mean. Repeated-measure ANOVA was used to produce *p*-values. *p < 0.05, **p < 0.01, ***p < 0.001, #*p* < 0.1

To investigate the hemodynamic impact of different peak IABP and peak MCC time differences, the effects of optimal ($\Delta {t}_{\mathrm{optimal}}$), least optimal, unfavorable $\Delta t$ ($\Delta {t}_{\mathrm{unfav}.}$), and baseline MCC were compared. MAP and mCBF were significantly higher at $\Delta {t}_{\mathrm{optimal}}$ compared to baseline (*p* < 0.01 for both variables) and $\Delta {t}_{\mathrm{unfav}.}$ (*p* < 0.01 and *p* < 0.001 for MAP and mCBF, respectively), and significantly higher at baseline compared to $\Delta {t}_{\mathrm{unfav}.}$ (*p* < 0.05 for both, Fig. 3a,c). Similar differences were found for mJBF, with the increased flow for $\Delta {t}_{\mathrm{optimal}}$ compared to $\Delta {t}_{\mathrm{unfav}.}$ and baseline, with the latter showing a trend toward being higher than $\Delta {t}_{\mathrm{unfav}.}$ (*p* < 0.01 for $\Delta {t}_{\mathrm{optimal}}$ vs. MCC, *p* < 0.01 for $\Delta {t}_{\mathrm{optimal}}$ vs. $\Delta {t}_{\mathrm{unfav}.}$, *p* < 0.1 for MCC vs. $\Delta {t}_{\mathrm{unfav}.}$; data not shown). For CPP, no difference was observed between $\Delta {t}_{\mathrm{optimal}}$ and baseline, while there was a trend toward decreased CPP when comparing baseline to $\Delta {t}_{\mathrm{unfav}.}$ (*p* < 0.1) and a statistically significant decrease when comparing $\Delta {t}_{\mathrm{optimal}}$ to $\Delta {t}_{\mathrm{unfav}.}$ (*p* < 0.05) (Fig. 3b).

**Fig. 4:**
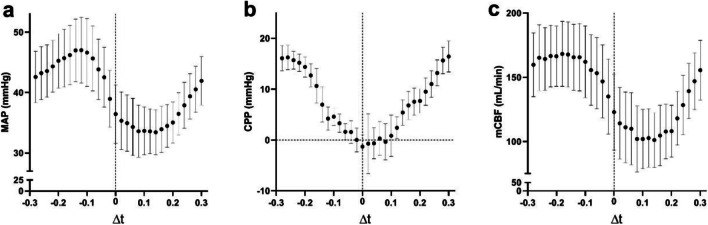
MAP (**a**), CPP (**b**), and mCBF (**c**) for all $\Delta t$ during MCC with IABP in anesthetized pigs. Bars and whiskers denote mean values ± SE. Each individual data point is aggregated from 6 animals, with each animal contributing 8–12 samples per $\Delta t$

Analysis of the range of $\Delta t$ for IABP animals revealed a pattern where the highest MAP occurred around $\Delta t$ between $-$ 0.16 and $-$ 0.08 s, *i.e.*, when IABP peak pressure was reached 0.08 to 0.16 s before MCC peak pressure (Fig. 4a). For mCBF, the optimal inflation timing in relation to MCC appeared more permissive; optimal mCBF was achieved when $\Delta t$ was between $-$ 0.26 and $-$ 0.12 s (Fig. 4c). In contrast to MAP, the optimal timing for maximizing CPP was at $\Delta t$ between 0.26 and 0.24 s, corresponding to IABP inflation late in the current MCC cycle (or early in the next MCC cycle).

### Intra-caval Balloon Pump Experiments

#### $\Delta {{t}}$ for ICBP and MCC

**Fig. 5:**
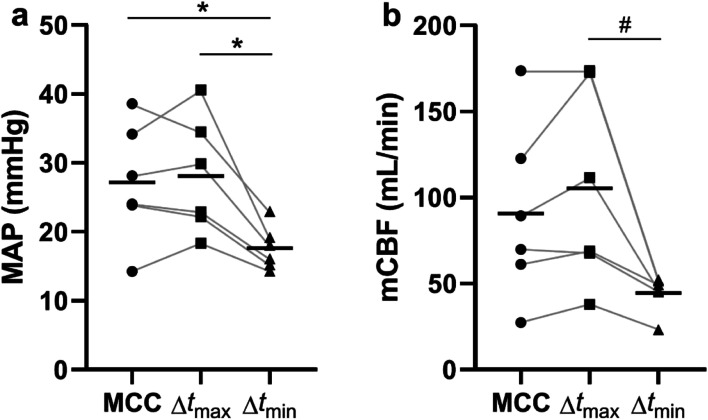
MAP (**a**) and mCBF (**b**) at baseline (MCC) without concurrent ICBP, optimal $\Delta t$ ($\Delta {t}_{\mathrm{optimal}}$), and least optimal $\Delta t$ ($\Delta {t}_{\mathrm{unfav}.}$), in anesthetized pigs. Bars denote the sample mean. Repeated-measure ANOVA was used to produce *p*-values. #*p* < 0.1, *p < 0.05

The optimal timing of phase shift between ICBP balloon peak pressure and MCC peak pressure was explored, analogous to the analysis performed for IABP and MCC above. MAP was significantly higher at $\Delta {t}_{\mathrm{optimal}}$ compared to $\Delta {t}_{\mathrm{unfav}.}$ (*p* < 0.05) but not compared to baseline, and significantly lower at $\Delta {t}_{\mathrm{unfav}.}$ compared to baseline (*p* < 0.05, Fig. 5a). For mCBF, there was a trend toward decreased blood flow at $\Delta {t}_{\mathrm{unfav}.}$ compared to $\Delta {t}_{\mathrm{optimal}}$, (*p* < 0.1; Fig. 5b). No differences were seen when comparing mCBF at baseline to $\Delta {t}_{\mathrm{optimal}}$ and $\Delta {t}_{\mathrm{unfav}.}$. For CPP, $\Delta {t}_{\mathrm{optimal}}$ and $\Delta {t}_{\mathrm{unfav}.}$ could not be readily identified, possibly due to ICBP interfering with CVP measurements.

**Fig. 6:**
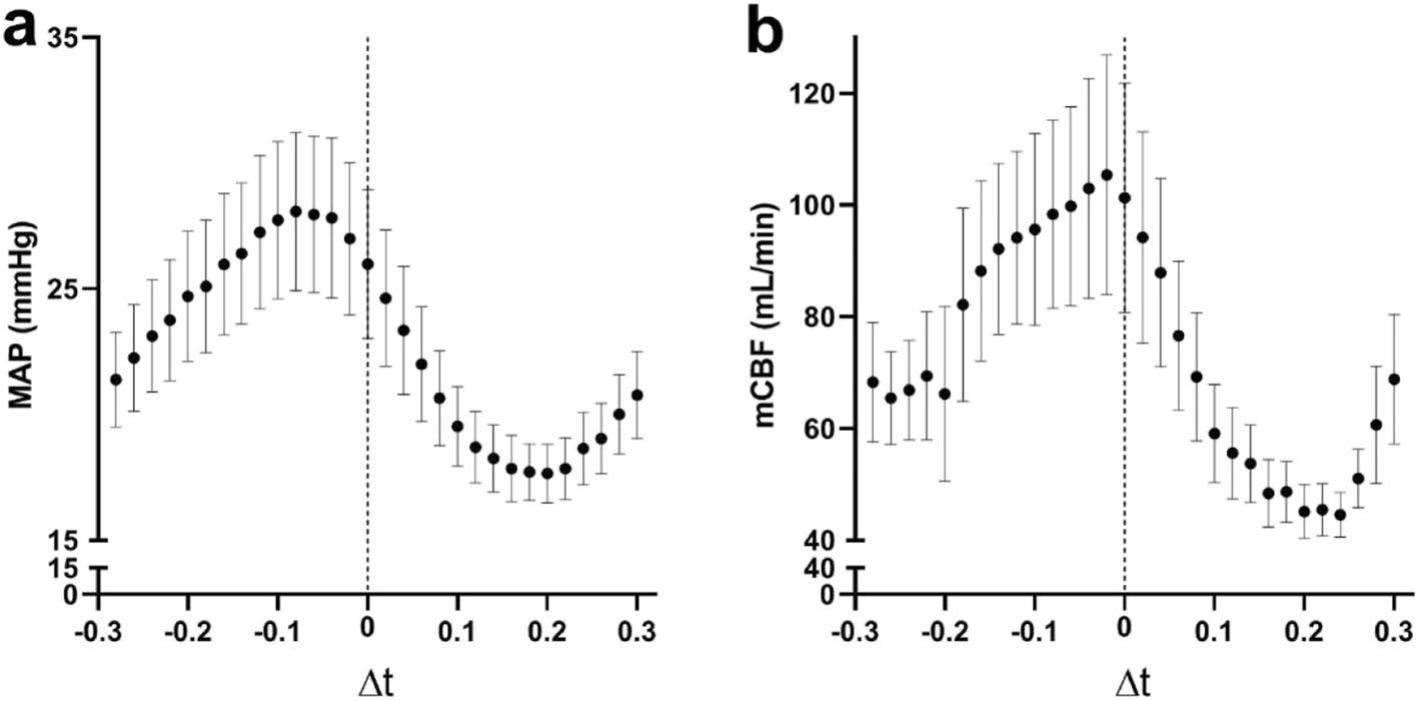
MAP (**a**) and mCBF (**b**) for all $\Delta t$ during MCC and ICBP in anesthetized pigs. Bars and whiskers denote mean values ± SE. Each individual data point is aggregated from 6 animals, with each animal typically contributing 8–12 samples per $\Delta t$

The highest mean MAP was achieved at $\Delta t$ between $-$ 0.10 and $-$ 0.04 s (ICBP peak balloon pressure occurred 0.04 to 0.10 s before MCC peak pressure; Fig. 6a). For mCBF, there was a wider array of $\Delta t$ showing increased mean mCBF, with $\Delta t$ between $-$ 0.16 and 0.0 s (Fig. 6b).

## Discussion

To the best of our knowledge, this is the first study to examine the optimal timing for IABP in the CPR cycle. It is also the first study to evaluate the effects of ICBP during CPR. The main results showed that the hemodynamics were affected by the timing of IABP and ICBP inflation. MAP, mCBF, and mJBF all increased significantly when IABP inflated prior (~ 0.15 s) to the peak of MCC (SAP), *i.e.*, inflated during MCC compression, compared to when being inflated after MCC peak pressure (~ 0.1 s after) and to MCC only. CPP was significantly higher when IABP inflation occurred ~ 0.25 s prior to MCC peak pressure, corresponding to inflation during MCC decompression, compared to inflation at MCC peak pressure. No significant changes were seen during IABP, when CPP was compared to MCC only. MAP and mCBF were significantly increased when ICBP was inflated just prior to MCC peak pressure compared to after; no significant changes were seen when compared to MCC only, indicating that manipulation of venous pressures and blood flows in this manner during MCC may not be beneficial.

The objective of CPR is to sustain efficient cardio-cerebral perfusion to enhance the chances of ROSC with a favorable outcome. Direct and reliable measurements of the cerebral and coronary perfusions during CPR have been difficult to standardize due to the complexity of flow and pressure changes during chest compression and decompression compared to spontaneous circulation; surrogate measures of effective CPR have therefore been used. In our study, MAP, CPP, and mCBF were used as measures of cardiac output and cardio-cerebral perfusion.

CPP is believed to be one of the primary driving forces of myocardial blood flow during CPR. It has been suggested as a marker of effective CPR and is dependent on the DBP and CVP [5, 28]. During CPR, it is believed that a retrograde direction of coronary blood flow occurs during compression and that the heart is perfused mainly during the decompression phase [29]. In our study, maximum CPP was observed when the IABP was inflated during MCC decompression, 0.25 s before MCC peak pressure, probably following the principles of “diastolic augmentation” seen when IABP inflates during diastole in spontaneous circulation [16]. However, MAP and mCBF, markers of effective output, increased significantly when the IABP was inflated 0.15 s prior to MCC peak pressure, thus being inflated during chest compression, plausibly explained by centralization of the blood volume, analogous to the situation with REBOA [10]. The reason for these outcomes could be the correlation to the opening and closure of the aortic valve. In spontaneous circulation, too early inflation of the IABP causes premature closure of the aortic valve. This impairs the left ventricular ejection and increases myocardial oxygen demand [30, 31]. In our study, the most unfavorable hemodynamics were observed when IABP was inflated 0.1 s after MCC peak pressure; thus, inflation occurred when the aortic valve was, most likely, open, impairing forward flow. Therefore, inflation of IABP during MCC should occur when the aortic valve is closed. The CPP obtained with optimally timed IABP inflation did not reach statistical significance when compared to MCC only. However, the highest CPP was seen when IABP inflation occurred 0.25 s prior to MCC peak pressure, and MAP and mCBF increased significantly when IABP inflation occurred 0.15 s prior to MCC peak pressure. It is likely, therefore, that the best cardio-cerebral perfusion occurs when the IABP inflates around 0.2 s prior to MCC peak pressure.

In cardiac arrest, IABP differs from REBOA in some aspects. It is likely that REBOA causes ischemic insult to the organs distal to the occlusion, further aggravating an already ischemic condition. An IABP could, therefore, be beneficial by permitting intermittent distal blood flow. Furthermore, during MCC, afterload is overcome by the external force of compressions. However, if ROSC is achieved, a totally occluding REBOA increases afterload greatly, thereby increasing the myocardial workload and oxygen demand [32] with the risk of reoccurring cardiac arrest. REBOA should therefore be deflated as soon as possible, although prompt REBOA deflation could be detrimental due to a pronounced decrease in arterial blood pressure and, subsequently, a decrease in CPP [33]. With IABP, the inflation-deflation timing during MCC could be reverted to “clinical settings” when ROSC is achieved, with decreased afterload and myocardial oxygen demand.

Essential for increasing ventricular outflow is to increase systemic venous inflow. Manipulation of preload has been shown to improve outcomes in cardiac arrest patients [34]. In our study, an ICBP was used in an attempt to increase preload. When the ICBP inflated just prior (0.1 s) to MCC peak pressure, during compression, significantly higher values were seen for MAP, and a trend toward higher values was seen for mCBF, compared to when the ICBP inflated after (0.2 s) MCC peak pressure, *i.e.*, being inflated during MCC decompression. No significant changes were seen when optimally timed ICBP values were compared to MCC, only indicating a lack of improvement with ICBP during CPR.

This study has some limitations. The duration of cardiac arrest (10 min) may impair the cardiovascular system and influence the hemodynamic effect of the interventions, although this time was chosen to reduce the frequency of ROSC. Another limitation was the use of healthy young pigs, and this may influence the transferability of the results to adult patients with cardiovascular disease. The study was not designed to evaluate the effect on ROSC of IABP/ICBP. The aim was to study the hemodynamic effects on different timings in correlation to the MCC. Our study did not, therefore, define the exact timing for when the IABP should be inflated with regard to ROSC.

## Conclusions

Manipulation of forward and backward pressures and flow, and preload, clearly affected hemodynamic variables during CPR. IABP significantly enhanced MAP and mCBF if inflation of the balloon occurred before the peak pressure of the MCC, whereas ICBP did not improve hemodynamic variables when compared to CPR alone. This study shows the potential of IABP in CPR when optimally synchronized with MCC and that further experimental studies are warranted.

## Supplementary Information

Below is the link to the electronic supplementary material.

Supplementary file1 (DOCX 78 KB)

## Abbreviations

CBF: Carotid blood flow
mCBF: Mean carotid blood flow
CPP: Coronary perfusion pressure
DBP: Diastolic blood pressure
IABP: Intra-aortic balloon pump
ICBP: Intra-caval balloon pump
LUCAS: Lund university cardiac assist system
MAP: Mean arterial blood pressure
MCC: Mechanical chest compression
REBOA: Resuscitative endovascular balloon occlusion of the aorta
ROSC: Return of spontaneous circulation
SAP: Systemic arterial blood pressure
VF: Ventricular fibrillation
