# Supplementary material for: Intra-aortic and Intra-caval Balloon Pump Devices in Experimental Non-traumatic Cardiac Arrest and Cardiopulmonary Resuscitation
Source: J Cardiovasc Transl Res. 2022 Dec 8;16(4):948–55. doi: 10.1007/s12265-022-10343-9 (PMC10480270; doi:10.1007/s12265-022-10343-9)
Supplement: Supplementary file 1 — Supplementary file1 (DOCX 78 KB) [file 12265_2022_10343_MOESM1_ESM.docx]

# Supplementary: Hemodynamic parameters over time with MCC and IABP

For animals with IABP and MCC, a decline in parameters over the time period was observed during data acquisition. A regression model was fitted to normalized mean SBP over the duration with MCC and IABP, revealing a significant negative association (R^2^=0.25, p<0.0001). For ICBP, also analyzing normalized mean SBP, the association was also significant (p<0.0001), albeit with a lower correlation coefficient (R^2^=0.02) (**Fig S1**). Comparing the fitted regression models to the null model, *i.e*. the sample mean, the IABP model, taking the declining trend into account, was a better fit to the data expressed as modified AIC ($AIC=n log(SSE/n)+2k +1$, SSE: summed square error, *n*: number of time points, k: number of parameters in model) (${AIC}_{model}=-1007.4$ vs. ${AIC}_{null}=-752.0$ for IABP compared to ${AIC}_{model}=-1406.0$ vs. ${AIC}_{null}=-1385.2$ for ICBP).


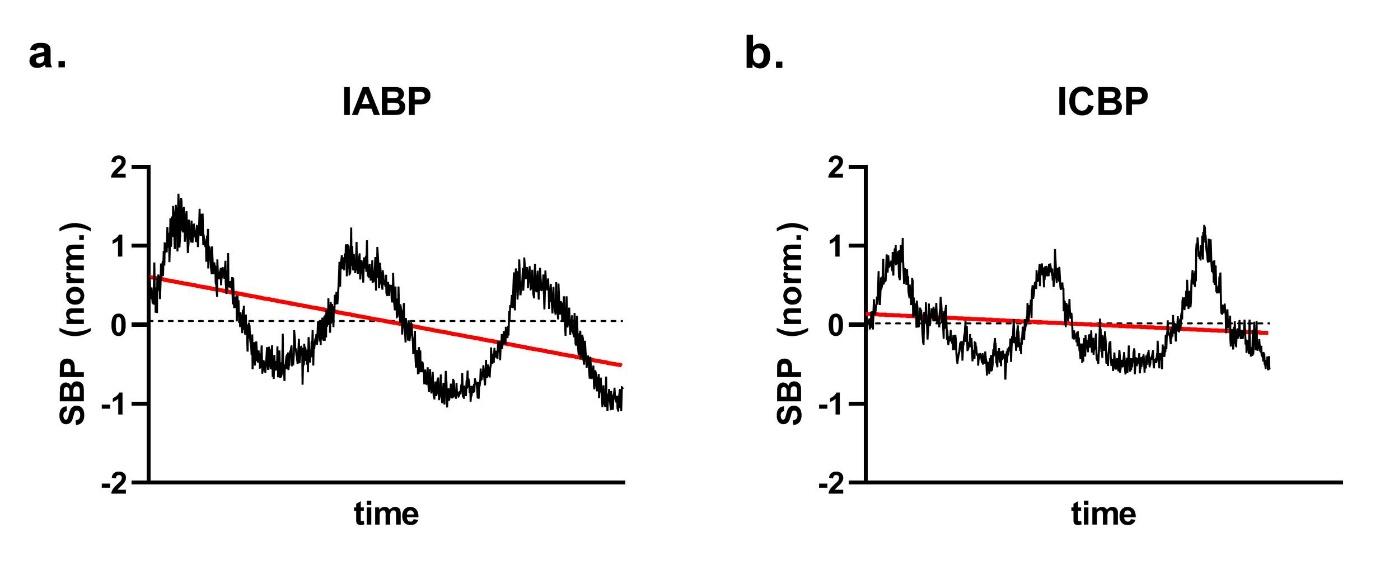


**Fig. S1** Typical normalized mean SBP during MCC with IABP (left) or ICBP (right). ▬: normalized SBP, ▬: fitted regression model, ▬ dashed: null model (sample mean)

To be able to compare absolute, non-normalized, pressure and mean flow values between different $\Delta t$, parameter data from the MCC+IABP/ICBP duration selected ensuring that all values of $\Delta t$ were represented. Using the frequencies of MCC and IABP/ICBP, the beat frequency of wave superposition was 0.00525 Hz, yielding a beat time period of 190.6 s. By defining the collection time period to at least the beat time period, all $\Delta t$ were collected. In effect, this corresponds to roughly a third of the full CPR+IABP/ICBP duration, as can be seen in **Fig S1**, left panel.
